# HCV and kidney transplant in the era of new direct-acting antiviral agents (DAAs)

**DOI:** 10.1007/s40620-018-0476-4

**Published:** 2018-02-06

**Authors:** Gaetano La Manna

**Affiliations:** Department of Experimental, Diagnostic and Specialty Medicine (DIMES)-Nephrology, Dialysis and Transplantation Unit, St. Orsola Hospital, University of Bologna, Via G. Massarenti 9 (Pad. 15), 40138 Bologna, Italy

The prevalence of patients with end-stage kidney disease (ESKD) on hemodialysis affected by hepatitis C virus (HCV) infection is 7.5% and many of these patients are on the waiting-list for kidney transplant [1]. Serum HCV positivity in kidney transplant recipients is significantly higher (around 10%) than in the general population, mostly due to the history of frequent blood transfusions, and it is related to the time on hemodialysis prior to transplantation [2]. HCV infection in renal transplant recipients is associated with increased morbidity and mortality rate and it is responsible for both hepatic and extra-hepatic complications. Chronic hepatitis and hepatocellular carcinoma are the main form of liver disease after renal transplant, while fibrosing cholestatic hepatitis, even if serious, has a more limited frequency [2, 3]. Extra-hepatic disease includes recurrent or de novo HCV-related glomerular disease, transplant glomerulopathy, acute rejection and a direct and/or indirect effect on kidney fibrosis [2, 3]. These complications usually occur earlier in HCV positive renal transplant recipients than in the negative ones, in particular transplant glomerulopathy, a condition of glomerular lesions specific to renal transplant characterized by specific histopathologic findings: at light microscopy a double contouring of glomerular basement membranes with no evidence of immune deposits, which can be detected 1 month after transplant with electron microscopy [4]. The association between HCV infection and reduced graft survival in kidney transplant recipients has still to be completely clarified and there are many factors potentially involved. Several data have demonstrated how hepatic and extra-hepatic complications after kidney transplant are responsible for reduced graft survival. Recent papers have shown that HCV positive (HCV+) patients with kidney transplant have reduced graft survival compared to HCV negative (HCV−) patients [2, 5]. Among the extra-hepatic complications, new onset diabetes after transplant, infection and cardiovascular disease are more frequent in HCV+ patients and are correlated with higher mortality [3, 5].

In 2011, the U.S. Food and Drug Administration (FDA) approved the introduction of direct-acting antiviral agents (DAAs) for the treatment of chronic HCV. DAAs consist in a group of small molecules that target the nonstructural viral proteins NS3, 4A, 4B, 5A and 5B. The administration of DAAs represents a new and effective treatment option also for ESKD and transplant patients affected by HCV infection. Up until a few years ago, the management and treatment of HCV+ patients with ESKD and kidney transplant posed a real challenge. Indeed classical treatment of HCV infection with an interferon-based regimen was associated with poor tolerability, low efficacy and unacceptably high rates of acute kidney injury, acute rejection and graft failure [5].

Currently, practice guidelines by the European Association of the Study of the Liver (EASL) [6], the American Association for the Study of Liver Disease (AASLD) and the Infectious Diseases Society of America (IDSA) [7] suggest, for patients with mild to moderate renal failure and ESKD patients affected by HCV genotypes 1 and 4, infection treatment with elbasvir/grazoprevir and ombitasvir/paritaprevir/ritonavir or ombitasvir/paritaprevir/dasabuvir plus or minus ribavirin, while effective practice guidelines for the treatment of the remaining genotypes are still lacking. Recently, a multicenter phase-3 study (EXPEDITION-4 trial) [8] evaluated the treatment with glecaprevir/pibrentasvir regimens for infection by HCV genotypes 1, 2, 3, 4, 5 or 6 in 104 patients with chronic kidney disease (CKD) stage 4 and 5 (82% on hemodialysis), and a sustained virologic response was achieved in 98% of the treated patients, with no drug-related serious adverse events.

Even more so than in ESKD, the experience with DAAs in the treatment of HCV+ kidney transplant recipients is poor and definitely even more limited than in liver transplant recipients. In a recent multicenter randomized controlled trial, Colombo et al. [9] enrolled 114 kidney transplant recipients (median estimated glomerular filtration rate, eGFR, 56 ml/min) with HCV infection genotypes 1 and 4. Ledipasvir/sofosbuvir were administered for 12 or 24 weeks. All patients achieved a sustained virologic response at 12 and 24 weeks. A treatment-related reduction of eGFR was reported in 3 patients.

Recent data from the literature, even though heterogeneous, obtained on more than 300 kidney transplant recipients HCV+ treated with sofosbuvir-based regimens, confirm its efficacy in maintaining a sustained virologic response at 12 months, without serious adverse events reported [9]. The issue of drug interactions in kidney transplant patients is always a crucial point to keep into consideration, in this case between DAAs and immunosuppressive drugs: Colombo et al. [9] reported that just 18% of treated patients required a dose adjustment of calcineurin inhibitor without any consequent graft rejection.

Summarizing, DAAs treatment could create a revolutionized scenario in the field of ESKD patients and kidney transplant: eradication should become the rule rather than the exception, as suggested by current guidelines, with the aim to reduce mortality and morbidity of these patients as well as lower the risk of nosocomial infection [5–7]. Unfortunately, as reported in the DOPPS study (Dialysis Outcomes and Practice Patterns Study) [1], in everyday clinical practice less than just 2% of patients in dialysis and less than 5% of patients wait-listed for kidney transplant are treated for HCV eradication. A possible reason why dialysis patients are not treated before transplant is the eventual advantage of obtaining grafts from HCV+ donors, with consequent shortening of the waiting-list time: this could be useful in countries such as the United States, where HCV+ donors are more than 20% [5]. However, this theoretical benefit would be lost in countries where the percentage of HCV+ donors is much lower, such as in European countries.

Therefore, HCV eradication after kidney transplantation should ideally be done “as soon as possible”, even if the eligible time for starting the treatment is still far from being well established, because the beginning of immunosuppressive therapy could create adverse events, mostly related to antiviral-treatment complications. As previously reported, Colombo et al. described a few cases of GFR reduction in kidney transplant recipients treated with DAAs; therefore a close monitoring of renal function is required, with the aim to early detect any graft function impairment [9]. Recent promising studies have reported that the expression of some protein biomarkers, such as neutrophil gelatinase-associated lipocalin (NGAL), a molecule with multiple activities, including anti-inflammatory and immunomodulatory effects, can provide information able to early detect possible renal damage [10, 11].

Furthermore, the efficacy and safety of DAAs would offer the opportunity to allocate kidneys from HCV+ donors to HCV− patients, as reported in the THINKER trial [12], where 10 allografts from HCV genotype 1 viremic donors were allocated to HCV-uninfected recipients. They were successfully treated with a 12-week regimen of elbasvir/grazoprevir and all patients achieved a stable virologic response and renal function.

In conclusion, DAAs have dramatically improved the cure rate for HCV infection treatment. This opportunity should now be extended to the field of kidney transplant, where it can open up new positive and safe prospects for both ESKD populations wait-listed for kidney transplant and patients with renal graft affected by HCV infection. These data need to be confirmed in long-term studies.
